# Automatic motion compensation for structured illumination endomicroscopy using a flexible fiber bundle

**DOI:** 10.1117/1.JBO.25.2.026501

**Published:** 2020-02-25

**Authors:** Andrew D. Thrapp, Michael R. Hughes

**Affiliations:** University of Kent, School of Physical Sciences, Applied Optics Group, Canterbury, United Kingdom

**Keywords:** endomicroscopy, structured illumination, motion compensation, mosaicking, digital micromirror device

## Abstract

**Significance**: Confocal laser scanning enables optical sectioning in clinical fiber bundle endomicroscopes, but lower-cost, simplified endomicroscopes use widefield incoherent illumination instead. Optical sectioning can be introduced in these simple systems using structured illumination microscopy (SIM), a multiframe digital subtraction process. However, SIM results in artifacts when the probe is in motion, making the technique difficult to use *in vivo* and preventing the use of mosaicking to synthesize a larger effective field of view (FOV).

**Aim**: We report and validate an automatic motion compensation technique to overcome motion artifacts and allow generation of mosaics in SIM endomicroscopy.

**Approach**: Motion compensation is achieved using image registration and real-time pattern orientation correction via a digital micromirror device. We quantify the similarity of moving probe reconstructions to those acquired with a stationary probe using the relative mean of the absolute differences (MAD). We further demonstrate mosaicking with a moving probe in mechanical and freehand operation.

**Results**: Reconstructed SIM images show an improvement in the MAD from 0.85 to 0.13 for lens paper and from 0.27 to 0.12 for bovine tissue. Mosaics also show vastly reduced artifacts.

**Conclusion**: The reduction in motion artifacts in individual SIM reconstructions leads to mosaics that more faithfully represent the morphology of tissue, giving clinicians a larger effective FOV than the probe itself can provide.

## 1 Introduction

Histopathology is the current “gold standard” for the diagnosis of epithelial cancers. Typically, the process involves a clinician removing one or more tissue samples from a patient. The tissue is then fixed, sliced, and stained before being examined under a microscope. This lengthy process introduces difficulties for rapid diagnosis or interoperative surgical guidance. A less-invasive alternative, an optical biopsy obtained using a fiber bundle-based endomicroscope, allows real-time microscopic imaging in otherwise inaccessible hollow tissue tracts or deep within hollow organs.^1^

Conventional histology has the benefit of providing physical sectioning by thinly slicing the tissue, allowing it to be observed under a conventional microscope. In an optical biopsy, as there is no physical sectioning, light from out-of-focus planes degrades the resulting images.^2^ This out-of-focus contribution can be rejected by introducing optical sectioning. One common technique is to use a confocal pinhole to block out-of-focus light from returning to the detector;^3^^–^^5^ several commercial confocal endomicroscopy systems employ this approach such as the Mauna Kea Cellvizio^6^ (Paris, France) and Optiscan^7^ system (Victoria, Australia). These systems are both fiber-based, use lasers, and require fast, precise scanning mechanisms.^8^ The scanning is either performed via a miniaturized fiber scanner sitting at the distal tip of the probe or using conventional scanning mirrors sitting proximal to the probe. In the latter case, the scanning pattern is relayed to the tissue via a coherent fiber imaging bundle with typically up to 30,000 cores.

Alternatively, a low-cost nonscanning endomicroscope can be built using widefield LED illumination delivered directly to the tissue via a fiber bundle with an image collected by the same bundle and imaged onto a camera via fluorescence filters.^9^^–^^11^ The fiber has no distal optics and is always in direct contact with the tissue, thus the surface of the sample is essentially deformed to be in constant contact with the probe tip. Such a system has virtually no inherent optical sectioning, but this can be added using structured-illumination microscopy (SIM),^12^^–^^14^ requiring only a modification to the illumination optics to project periodic symmetric line patterns onto the bundle, and thus onto the tissue.

In SIM, a thin slice around focus is modulated by the illumination pattern while out-of-focus regions see a defocused pattern, which approximates uniform illumination. Three images are acquired, each with the modulation pattern shifted by $\frac{1}{3}$ of a period. The three images are then processed to recover an optically sectioned image.^15^^–^^17^ In the ideal case, we can express the modulation $s_{i}(x,y)$ as

$$s_{i}(x,y)=\frac{1}{2}[1+m\;\sin(\nu x+\phi_{i})],$$ (1)

where m is the modulation depth, which ranges from 0 (no modulation) to 1 (complete modulation), ν is the spatial frequency of modulation, chosen based on desired optical-sectioning strength, and $\phi_{i}=\frac{i2\pi }{3}$ is the phase of the modulation for i=1,2,3.^18^

Once the three raw images, $I_{1},I_{2},I_{3}$, have been acquired, the modulation is removed, and an optically sectioned image $I_{\mathrm{sim}}$ is obtained via three-phase demodulation:^18^

$$I_{\mathrm{sim}}=\sqrt{{\left(I_{1}-I_{2}\right)}^{2}+{\left(I_{1}-I_{3}\right)}^{2}+{\left(I_{2}-I_{3}\right)}^{2}}.$$ (2)

Endomicroscopes are used in several configurations, including through the working channel of an endoscope,^19^ as part of a robotic or mechanical system,^20^^,^^21^ or in freehand operation.^22^ These three configurations all tend to involve a probe that is constantly, or often, in motion. In some circumstances, motion is deliberately introduced. A conventional biopsy is typically around $3\times 3\;{\mathrm{mm}}^{2}$ in size, while a fiber endomicroscope has a typical probe diameter of <1  mm. This size difference can make it difficult for clinicians to get a broader view of the tissue morphology.^23^ To increase the effective field of view (FOV), and consequently the amount of clinically relevant information, it is possible to merge sequentially acquired images during motion into a mosaic.^23^^–^^27^

However, since SIM is a three-frame acquisition process, motion between acquisition frames makes the recovered optically sectioned images susceptible to artifacts. The artifacts appear as blurring or chopping, explaining why mosaicking in structured illumination endomicroscopy has not been previously demonstrated. The shift between raw images due to motion of the probe can be corrected using registration providing it is sufficiently small relative to the frame rate. However, the consequence of this is that the line pattern is then no longer correctly positioned in the three images.

We have previously reported provisional results for cases when the motion is known in advance and hence where the lines can be aligned parallel to the motion, keeping the line pattern correctly positioned when images are registered and shifted. Once images have been shifted, SIM images can then be reconstructed in an overlapping area with reduced artifacts.^28^ The usefulness of this approach, however, is limited as motion is not typically known in advance and any change in the direction of motion would require changing the orientation of the line pattern. We now report an automatic feedback mechanism to compensate for a probe moving in any direction. We also demonstrate real-time mosaicking for lens tissue paper and bovine tissue using mechanical translation and freehand probe operation.

## 2 Methods

The basic operational principle in SIM endomicroscopy involves placing the tip of a multicore fiber-optic bundle (30,000 cores) at the focus of a microscope objective, projecting a periodic pattern of lines through the bundle and onto a sample, shifting the line pattern 1/3 of a spatial period for two additional images, and processing the returning raw images. Various approaches have been used to generate the line pattern^12^^,^^29^^,^^30^ and similar to some previous reports, we use a digital micromirror device (DMD) (Texas Instruments DLP LightCrafter 3000). The automatic motion compensation technique we introduce has two components—image registration and pattern correction. Image registration is the process of determining the angle and magnitude of motion, while pattern orientation correction is the reorientation of lines parallel to the motion.

### 2.1 System Description

**Fig. 1:**
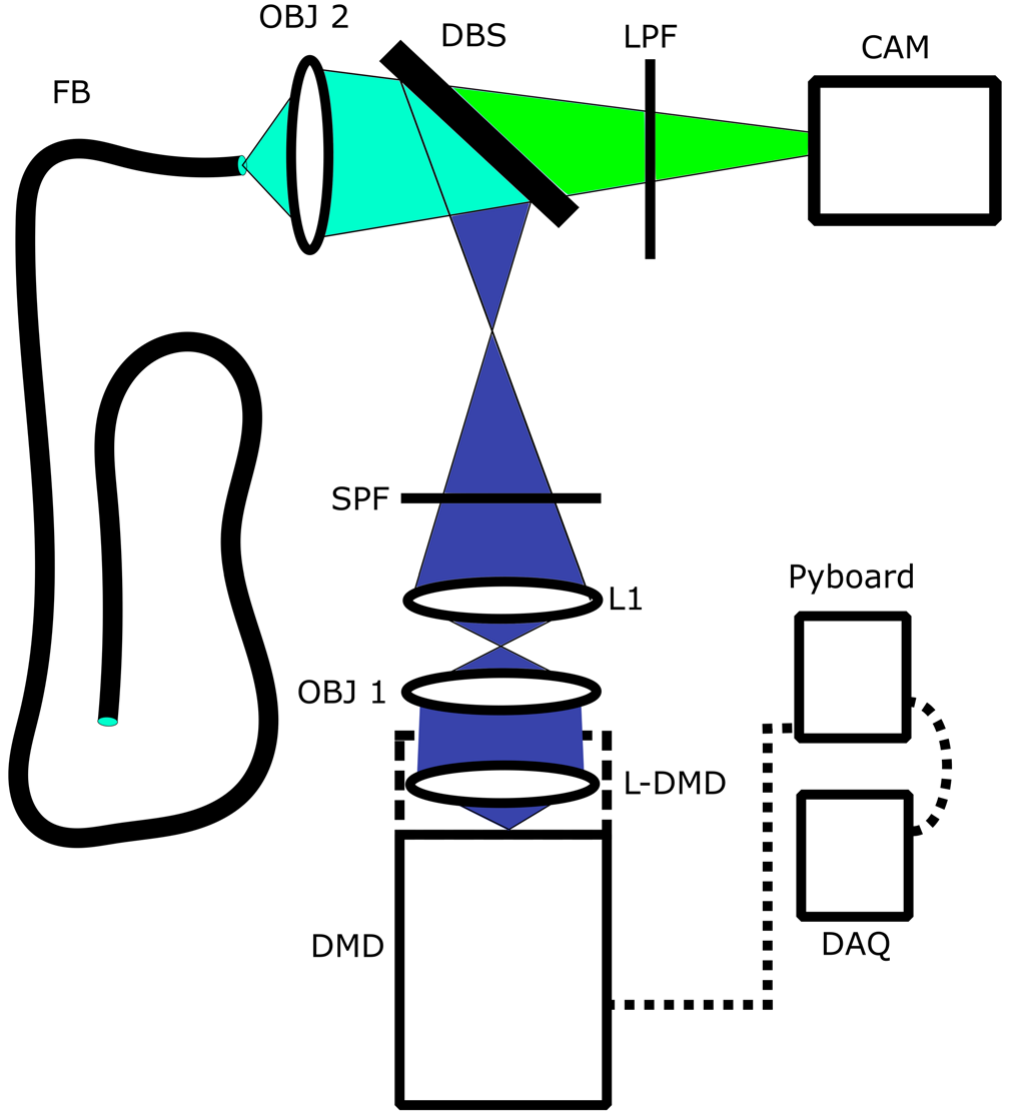
Schematic of endomicroscope. DMD, digital micromirror device; OBJ 1, 20× objective; OBJ 2, 4× objective; L1, 20 mm collimating lens; L-DMD, lens built into DMD; SPF, short-pass filter; LPF, long-pass filter; DBS, dichroic beam splitter; and FB, fiber bundle.

Light from the blue LED of the DMD’s built-in illumination system is directed through a 20×/0.25 NA finite-conjugate objective (Newport), a 20-mm collimating lens (Thorlabs ACL2520U), an excitation filter (Thorlabs FESH0450), and reflected off of a 490-nm cut-on dichroic mirror (Thorlabs DMLP490) onto the back aperture of a 4×/0.25 NA finite-conjugate objective. It is then transferred through a fiber optic bundle (Sumitomo IGN-08/30) in pixelated form. The returning fluorescence emission is imaged onto a camera (Point Grey Flea3) via the 4× objective, dichroic, and an emission filter (Thorlabs FELH0500). A diagram, showing the components as well as optical path, is presented in Fig. 1. A microcontroller (Pyboard 1.0) is used to control triggering of the camera and DMD (Fig. 3). A data acquisition (DAQ) card (NI USB 6008) provides communication between the controlling personal computer (PC) and the microcontroller. At the object plane, each camera pixel corresponds to $0.747\times 0.747\;\mu m^{2}$ with the 4-μm intercore spacing of the bundle, which provides better than Nyquist sampling. Each DMD pixel corresponds to $4.407\times 4.407\;\mu m^{2}$ measured at the object plane. The power at the fiber tip was measured to be 95  μW, and the fiber coupling losses were measured to be 89%. The system has no optics on the distal end of the fiber, meaning images are acquired in contact mode only.

**Fig. 2:**
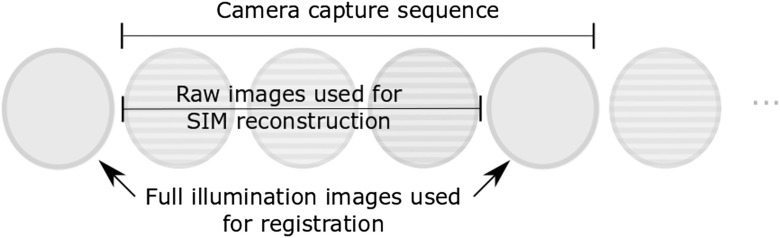
The four-frame acquisition sequence. The three SIM frames, with shifted line patterns, are book-ended by widefield illuminated frames used for image registration.

A custom-built LabVIEW (National Instruments) VI controls acquisition, registration, reconstruction, and pattern orientation. A summary of the program control loop is: (i) the Labview program sends the required pattern angle to the microcontroller via the DAQ card. (ii) The microcontroller selects the pattern orientation and triggers the necessary patterns on the DMD. (iii) A four-frame image sequence is acquired (Fig. 2) and a median filter is applied to the images. (iv) The angle and magnitude of motion between widefield illumination frames are determined by image registration using template matching. The shift is used for reconstruction and mosaicking, and the angle is used for the next update of the pattern orientation. (v) The raw frames are shifted to correct for motion between acquisition of each frame and an SIM image is reconstructed using Eq. (2). (vi) The reconstructed image is added into the mosaic.

### 2.2 Image Acquisition

Using the custom built VI, a four-frame sequence is acquired using LabVIEW image acquisition libraries (IMAQdx). This consists of three SIM raw images and one widefield (i.e., unstructured) illumination image (Fig. 2). The reason for introducing a widefield image is discussed in Sec. 2.3. Once images are captured, the honeycomb structure from the fiber bundle needs to be removed. There are several ways to accomplish this including two-dimensional (2-D) Gaussian filtering,^25^ spatial averaging,^31^ and core localization and interpolation methods.^23^ We filter the raw images using LabVIEW’s IMAQ Nth-Order filter as a median filter with a 4×4  pixel neighborhood ($3\times 3\;\mu m^{2}$)—pixel values are arranged by intensity in descending order and the central intensity value is selected.

### 2.3 Template Matching

**Fig. 3:**
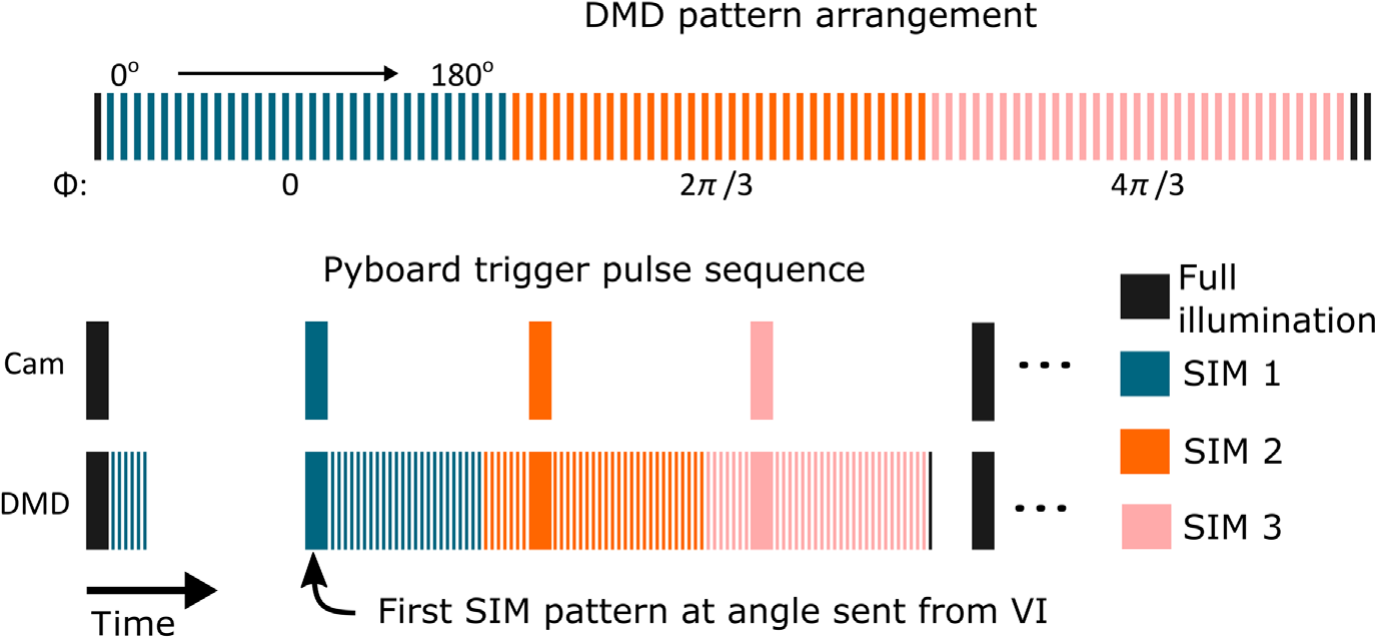
Pattern trigger structure. Top pane shows the sequence of patterns loaded onto the DMD. Each pattern must be triggered in order, but the exposure can be varied. Nonuniform pulses are sent to the DMD, with longer exposures for the required patterns synchronized with camera exposure trigger pulses, as shown in lower pane. The pattern is designed so that the camera receives a uniform pulse structure.

To determine the probe’s movement between image frames, we need to use template matching between overlapping frames. However, attempting to register two images with an overlapping grid pattern leads to incorrect registration when using a correlation-based approach. We instead register two widefield illumination images. Operationally, we accomplish this by adding one image to the end of the three-frame sequence and template matching the widefield illumination image from the current sequence with that of the previous (Fig. 2). Using an approach previously reported by Lewis for fast template matching,^32^ we extract a 250×250  pixel ($187.5\times 187.5\;\mu m^{2}$) square from the center of the current widefield illumination frame as a template, then calculate the normalized cross-correlation (NCC) matrix against the previous widefield illumination frame of 958×958  pixels ($718.5\times 718.5\;\mu m^{2}$). The estimated shift is then taken to be the location of the maximum peak in the NCC matrix. Since the pulse structure (Fig. 3) is designed to give uniformly spaced camera trigger pulses, we then assume uniform motion between each raw frame over the acquisition sequence and estimate the interframe shift between the raw SIM frames as 1/4 the shift between the widefield illumination frames. Finally, we shift the first and third SIM images relative to the second and generate optically sectioned reconstructions using Eq. (2).

### 2.4 Pattern Orientation Correction and Image Reconstruction

As discussed above, simply registering and shifting the images is insufficient to prevent artifacts since the line patterns are no longer correctly in phase. To reduce or eliminate the artifacts, the lines need to be oriented parallel to the motion of the probe.

The DMD can be used in several ways. A PC can output patterns to the DMD via a high-definition multimedia interface or patterns can be preloaded onboard. Onboard pattern storage was used to take advantage of the faster pattern switching possible with this approach and to avoid software lag when sending patterns over a video stream. We loaded patterns of different angles (0 deg to 180 deg) onto the DMD. The onboard storage supports storing 96 patterns, meaning 31 angles are supported (also allowing for a widefield frame), permitting a set of 3 patterns every 5.625 deg.

Patterns are selected by the microcontroller using the angle determined in template matching. Although the DMD does not allow us to select individual patterns at the hardware level, it does allow us to “skip” patterns after a minimum display time. As shown graphically in Fig. 3, the total acquisition time of a frame is equal to the exposure time ($t_{\exp}$) added to the product of the minimum display time ($t_{\min}$) and the maximum number of unwanted frames between SIM patterns ($n_{\text{skipped}}$). When fewer patterns are skipped, delays need to be introduced to keep the camera’s frame rate constant. The formula for the theoretical maximum raw frame rate (${\mathrm{fps}}_{\text{raw}}$) is then given by

$${\mathrm{fps}}_{\text{raw}}=\frac{1}{\left(t_{\exp}+n_{\text{skipped}}\times t_{\min}\right)}.$$ (3)

The reconstructed frame rate is then 1/4 of the raw frame rate. With 30 skipped frames, a ($t_{\min}$) of 325  μs, and an exposure of 1000  μs, this supports a theoretical maximum raw frame rate of 93 or 23.25 fps per reconstructed image. We acknowledge that this exposure is not ideal for many applications, including the acriflavine stained bovine stomach tissue issue images reported below, where we used an exposure of 25,000  μs. The theoretical maximum frame rate for this exposure duration is 28 fps raw frame rate or 7 fps per reconstructed image. For consistency, we operated at 10 or 2.5 fps reconstructed throughout all experiments reported here.

During template matching, the pattern angle is scaled by a factor of 180 and sent as an analog voltage using a DAQ (National Instruments USB-6008) to a Pyboard. The Pyboard converts the received voltage back to the angle and, using the pulse structure shown in Fig. 3, selects the appropriate angle of illumination pattern and outputs trigger pulses to the camera and DMD. During unwanted patterns, the Pyboard triggers the DMD every 325  μs and does not trigger the camera. To ensure the four frames are equally spaced in time, the camera must be triggered uniformly and additional delays are introduced in DMD triggering to achieve this.

Once a sequence has completed and the VI has received the final widefield illumination image in a sequence, pattern orientation is not updated instantaneously, which takes 135 ms for template matching and the required angle to be received by the Pyboard. At frame rates below 20 fps, this voltage update is received by the Pyboard during the next cycle and the pattern orientation is changed after that cycle has completed.

### 2.5 Mosaicking

In order to assemble a mosaic as the probe moves, the position of each reconstructed frame relative to the previous is determined from the registration between widefield images. The SIM reconstructed image is cropped to a user-selected circle and added dead-leaf into the mosaic, overwriting any previous pixel values.

### 2.6 Validating Uniform Linear Probe Motion

Our approach assumes probe positions, which in five-frame sets are uniformly spaced and approximately linear. To verify these two assumptions, we capture widefield images with a probe being translated across a fluorescently stained lens paper sample, register the images, and plot a probe trajectory. We take neighboring sets of five points and interpolate the ends. To verify uniformity, we report the average distance between frames as well as the mean of the absolute deviations. To verify linearity, we calculate the distance between the interpolated line and the probe position. We then calculate combined error by determining the location of three evenly spaced points on the interpolated line and calculate the distance from the actual probe positions. Frames are captured at 120 fps and we also downsample the data to obtain a similar interframe shift to Figs. 7 and 8. The results of this study are shown in Table 1.

**Table 1:** Translation stage and freehand probe deviation from uniform and linear motion. Dist, interframe distance; MD, mean deviation from uniform motion; ED, mean Euclidean distance from interpolated line; CE, mean combined error from predicted probe positions. TS, translation stage; FH, freehand; ds, downsampled. Neighboring sets of five coordinates. Values less than 6  μm are less than the resolution of the probe.

	Dist (μm)	MD (μm)	% MD	ED (μm)	% ED	CE (μm)	% CE
TS	19.3	0.9	4.4	0.6	2.9	1.2	6.1
${\mathrm{TS}}_{\mathrm{ds}}$	77.0	1.6	2.1	1.4	1.8	2.5	3.2
FH	8.6	1.1	16.8	0.3	4.0	1.4	21.2
${\mathrm{FH}}_{\mathrm{ds}}$	77.5	19.3	27.2	2.4	3.3	22.7	32.9

Using a translation stage with mean interframe shifts of 77.0 and 19.3  μm, the combined errors are, respectively, 3.2% (2.5  μm) and 6.1% (1.2  μm). In freehand with interframe shifts of 77.6 and 8.6  μm, the errors are, respectively, 32.9% (22.7  μm) and 21.2% (1.4  μm). Error is calculated in each five-frame set and averaged over all sets. With motion compensation, this predicts improvement in alignment in SIM reconstructions in all cases.

### 2.7 Line Spacing Selection

The line spacing is chosen based on a number of criteria. Since the line spacing determines the strength of the optical sectioning, the spacing must be small enough to sufficiently remove out-of-focus light,^33^ but large enough to minimize the effects of pixelation and discretization and for the approach to remain robust to motion.

**Fig. 4:**
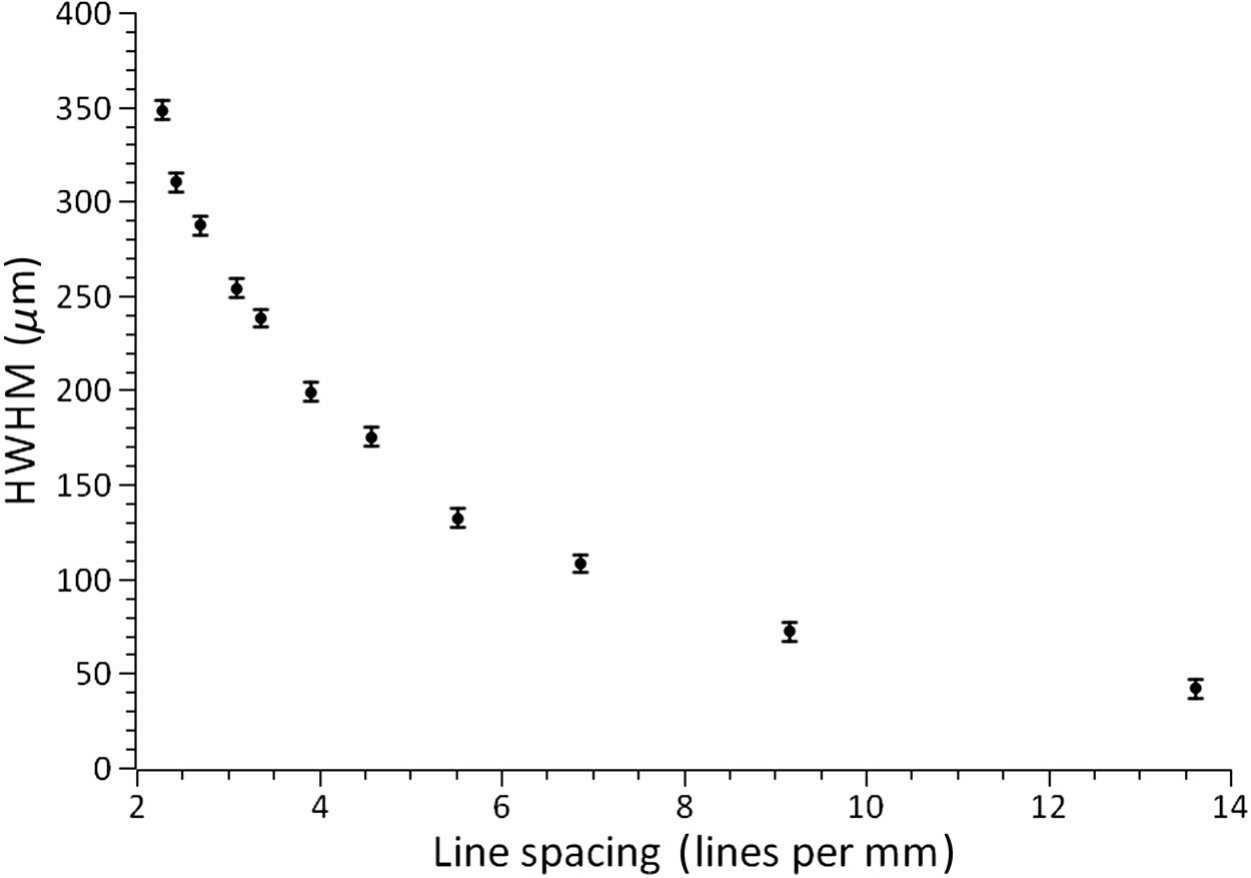
Half-width half-maximum of axial response as a function of line spacing. Data acquired using a smooth metal plate, fluorescently labeled with yellow highlighter, translated away from fiber bundle tip using a linear translation stage. Error bars reported as precision of stage (5  μm).

To determine how much out-of-focus light is rejected at each line spacing, we characterized the axial response of the system by measuring the relative intensity drop-off as a function of distance (Fig. 4). The study was performed by translating a smooth metal plate, stained with fluorescent marker, away from the tip of the fiber bundle at a known velocity using a translation stage (Newport M-UTM25PP1HL). The reconstruction intensity was then recorded as a function of distance by a custom-built LabVIEW VI.

Before being sent to the DMD, lines need to be discretized, leading to errors at the line boundaries, which can degrade reconstructions. Each DMD pixel is a square arranged on a grid and, when the lines are aligned with this grid, line spacings can be chosen with an integer number of pixels with common factors of 2 (to allow for half on and off mirrors), and 3 (to allow for three phase positions), eliminating discretization error. However, when the lines are rotated on the square grid, as required for the motion compensation approach described, this leads to discretization artifacts near the boundaries. Since these only occur at boundaries, the relative effect of this can be reduced at the expense of optical-sectioning strength by increasing the spacing of the lines (in pixels).

Even in cases where there is no discretization error, the fiber bundle is also susceptible to pixelation artifacts at the line boundary since the cores are laid out in a pseudohoneycomb structure.^34^ Selecting a line spacing much greater than the intercore spacing similarly reduces the number of boundaries and minimizes these artifacts at the expense of optical-sectioning strength.

**Fig. 5:**
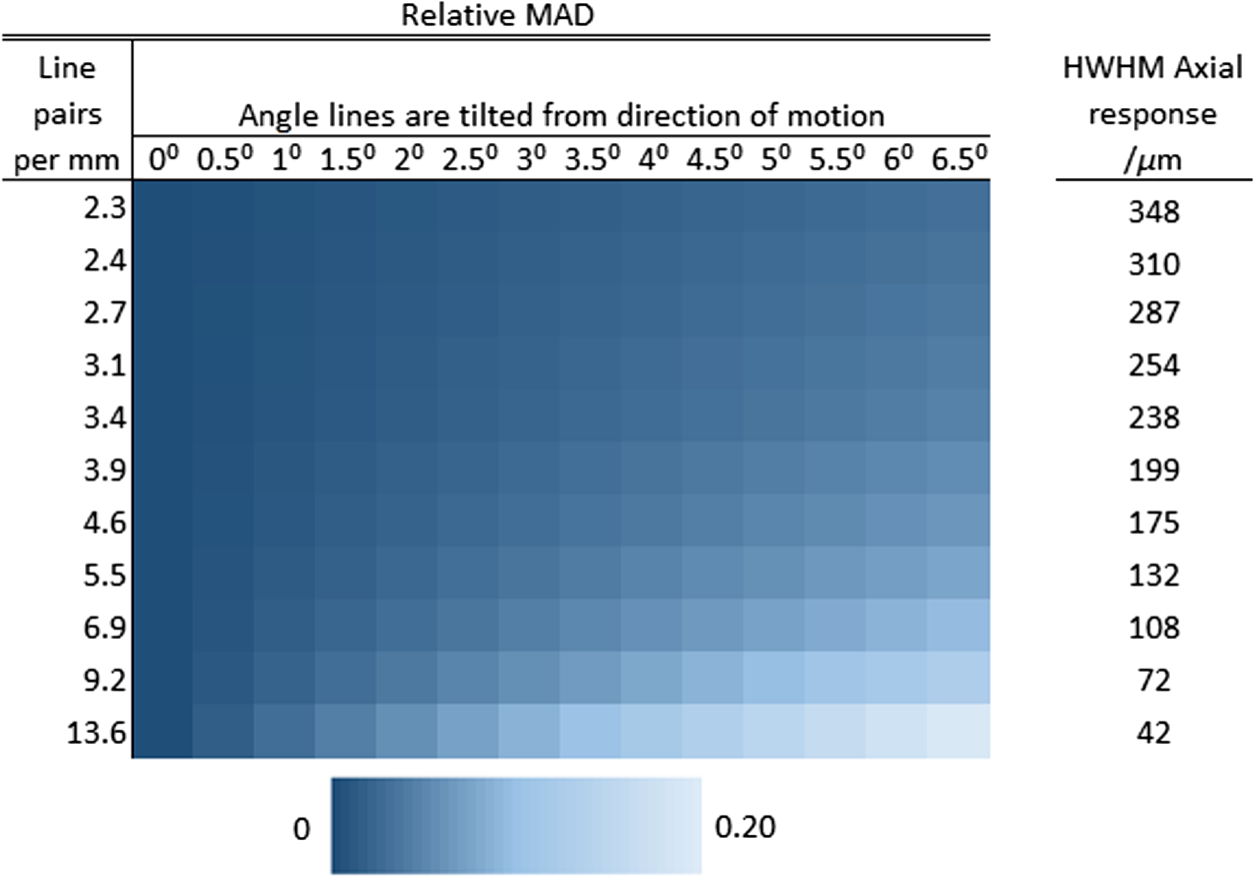
Simulation of SIM reconstructions when probe translation is not exactly parallel to the line orientation. The angle is measured between the direction of movement and line orientation. The plot shows relative MAD of reconstructions with different line spacings. Simulation assumed a 707  μm probe diameter and a 50  μm shift between frames.

Since the DMD only supports patterns every 5.625 deg, probe motion often introduces additional misalignment, thus the line spacing selected must generate reconstructions that are robust to probe motion when the motion is not exactly parallel to the line pattern orientation. This was characterized via a numerical simulation of SIM reconstructions with a shift between raw frames. In the simulation, a circle representing a probe in contact with a uniform single-layered, in-focus sample was superimposed with a line pattern of the form of Eq. (1). Three raw SIM frames were then acquired with the required phase shifts, with the probe also translated at some angle relative to the lines. The mean of the absolute differences (MAD) was then calculated between the moving SIM reconstruction and an ideal reconstruction in an overlapping area of the bundle. A 5000×5000  pixel grid was used, the modulation depth was assumed to be 1 (i.e., ideal), and pixelation effects from the fiber bundle and discretization effects from the DMD were ignored. The circle’s diameter was selected to match the fiber bundle (707  μm) and interframe shift (50  μm) used in the lens paper and bovine tissue studies below. The results showing the MAD as a function of line pair spacing and angular alignment error are shown in Fig. 5.

Based on the results shown in Figs. 4 and 5, we chose 6.9 line-pairs per millimeter (lp/mm) or a line pair spacing of 145  μm. Figure 4 shows that with lines at 6.9  lp/mm, out-of-focus light emanating from >108  mm from focus is heavily attenuated. The simulation using 6.9  lp/mm showed 95% similarity to static reference images (MAD=0.05) with an interframe shift of 50  μm and line pair orientation off-parallel by 3 deg. At this line spacing, pixelation artifacts are also minimal, and since the ∼30  pixels per line pair is much greater than the minimum 6 DMD pixels (4.5  μm) required for SIM, it is also much greater than the intercore spacing of the fiber bundle (3  μm). At this spacing, the modulation depth was measured to be 0.42 using the method reported by Hagen.^15^

## 3 Results and Discussion

**Fig. 6:**
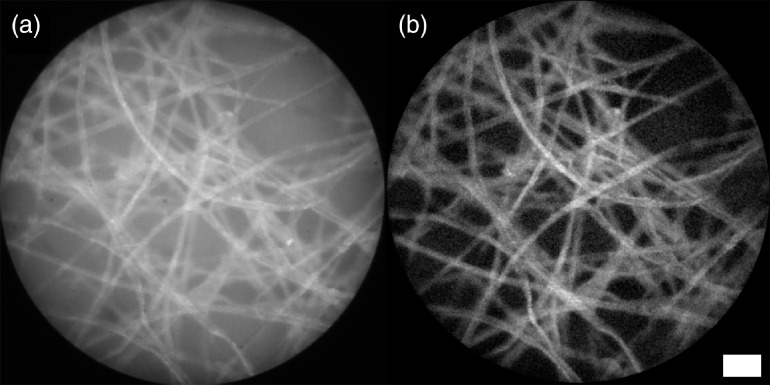
Fluorescently stained lens paper separated from a fluorescent background by a plastic sheet acquired with a stationary probe. Comparison of (a) widefield and (b) SIM images acquired with a fiber bundle probe. Bundle diameter: 707  μm. Line spacing: 6.4 lines per mm. Scale bar 100  μm.

To demonstrate the improvement of SIM endomicroscopy over widefield, we first mounted the probe to be stationary and in contact with a piece of lens paper stained with yellow highlighter. To simulate a fluorescent background signal, the lens paper was placed on top of a piece of paper also stained with highlighter, and the two were separated by a thin plastic sheet. Figure 6 clearly shows the effect of optical sectioning and the improvement in contrast.

**Fig. 7:**
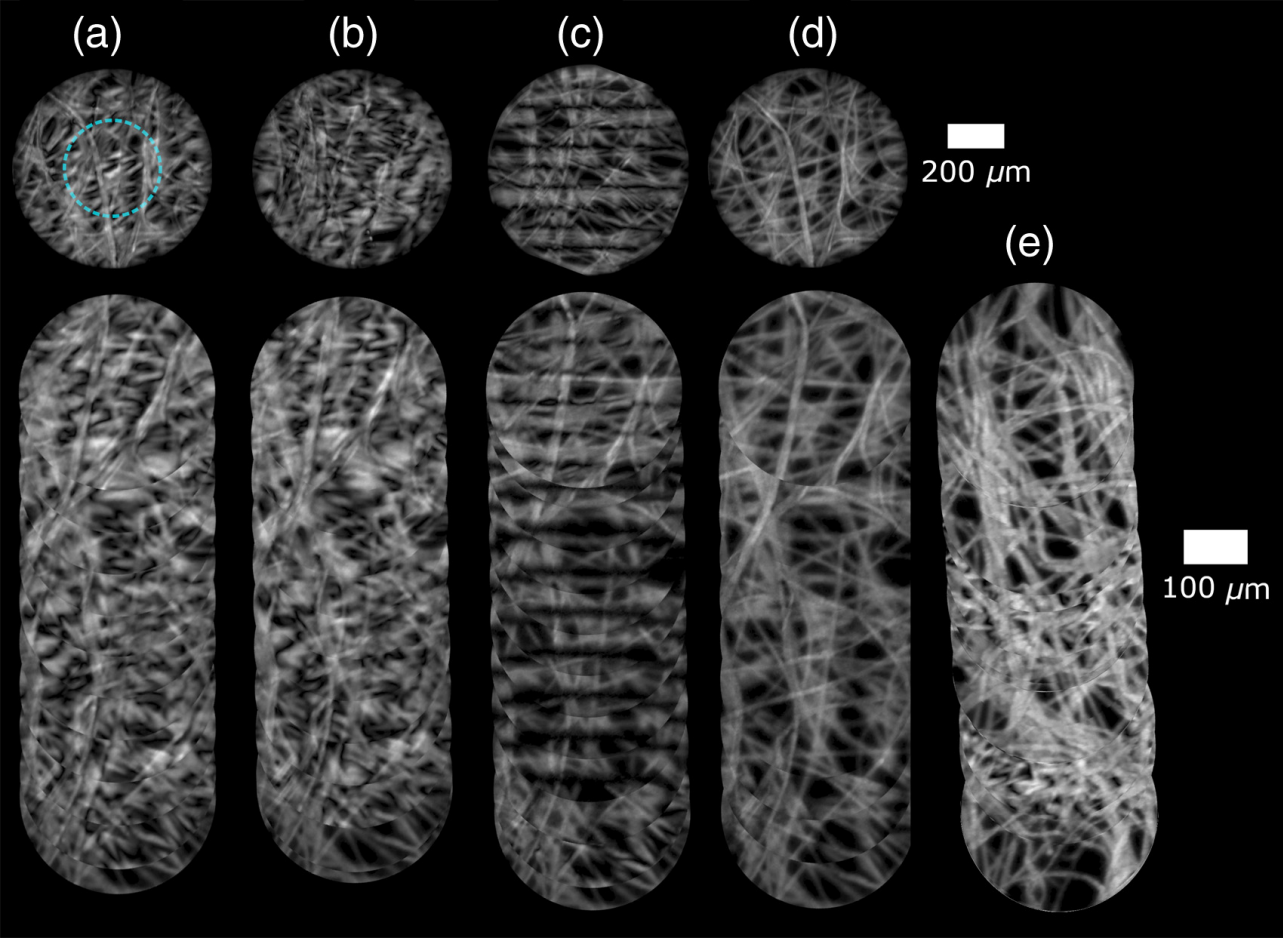
Moving probe SIM reconstructions and mosaics of lens paper stained with fluorescent highlighter. Lines at 6.9  lp/mm, frame rate of 2.5 fps per reconstructed image. Images (a)–(d) acquired with a translation stage (velocity was 0.25  mm/s). (a) Pattern correction off/image registration off, circle shows cropped area for mosaicking, (b) pattern correction on/image registration off, (c) pattern correction off/image registration on, (d) pattern correction on/image registration on, (e) freehand images—pattern correction on/image registration on. Cropped area used for mosaicking to eliminate residual lines near edges of reconstructions.

**Fig. 8:**
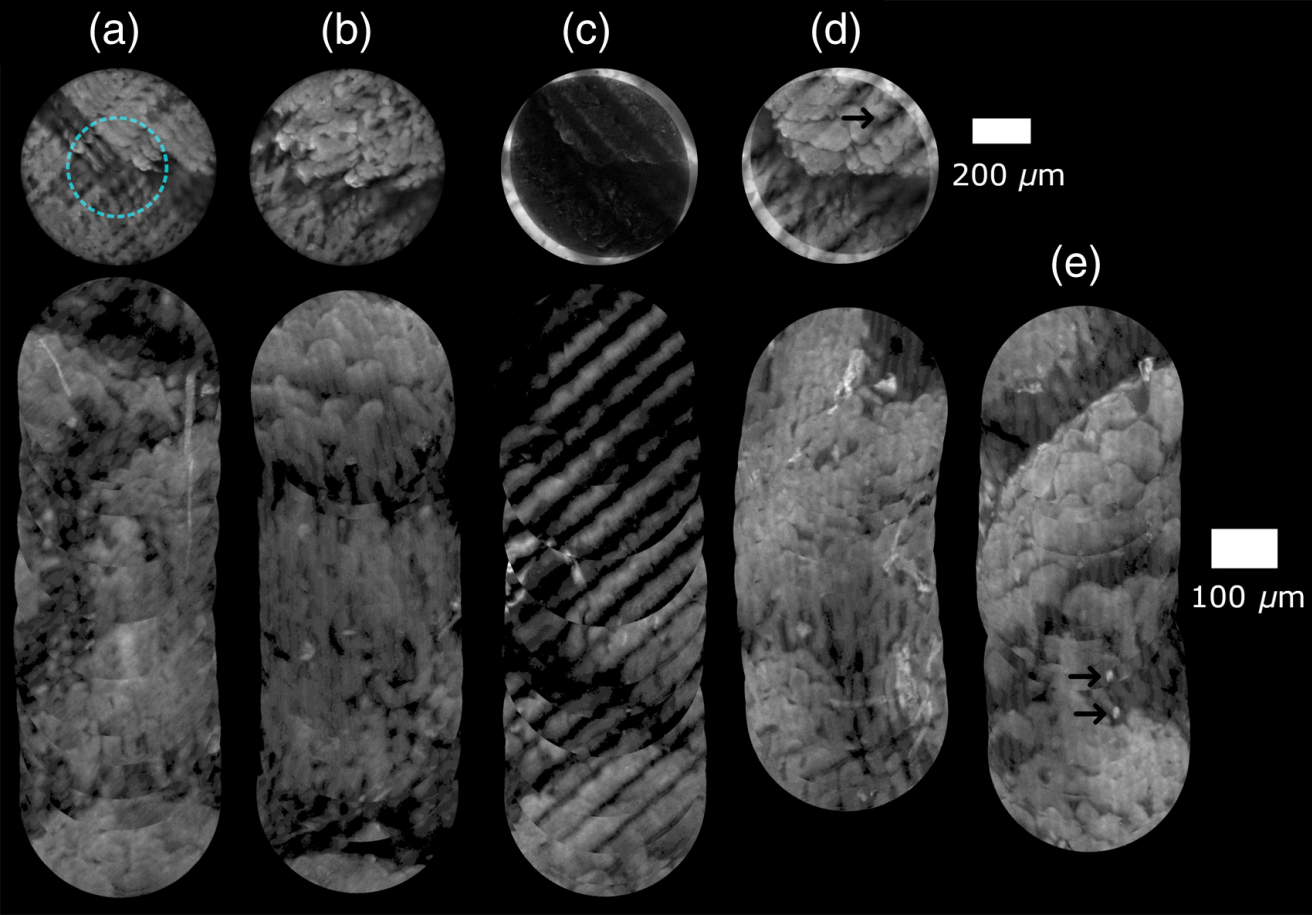
Moving probe SIM reconstructions and mosaics of bovine stomach labeled with acriflavine. Lines at 6.9  lp/mm, frame rate of 2.5 fps per reconstructed image. Images (a)–(d) acquired with a translation stage (velocity was 0.25  mm/s). (a) Pattern correction off/image registration off, circle shows cropped area for mosaicking, (b) pattern correction on/image registration off, (c) pattern correction off/image registration on, (d) pattern correction on/image registration on, (e) freehand images—pattern correction on/image registration on. Intensity values uniformly scaled by 2.5. Cropped area used for mosaicking to eliminate residual lines near edges of reconstructions.

To validate motion compensation with a moving probe, lens paper and bovine stomach were prepared for imaging. The lens paper was stained with yellow highlighter and the bovine tissue with acriflavine hydrochloride.^22^^,^^35^ The probe was placed in light contact with the sample, then translated across the sample at a velocity giving 50  μm between each raw image, corresponding to about 7% of the bundle’s diameter. This interframe shift, and hence velocity, was chosen as it is an example of a case of greatest degradation due to motion artifacts (certain velocities lead to less degradation if the interframe shift happens to be close to an integer multiple of the line spacing). The shift between the two widefield frames used for template matching was 200  μm, sufficiently small to provide an overlapping area for image registration. The maximum of the NCC as well as the relative MAD for the images in Figs. 7 and 8 are reported in Table 2. Examples of individual images are shown above the generated mosaics in Figs. 7 and 8.

**Table 2:** Relative MAD measurements as well as maximum of NCC matrix for lens paper and bovine stomach tissue. Obtained for lines at 6.9  lp/mm and with translation of 50  μm between raw images. Pat., pattern orientation correction and Reg., image registration.

	Pat. off, reg. off	Pat. on, reg. off	Pat. off, reg. on	Pat. on, reg. on
Lens paper				
NCC	0.44	0.39	0.97	0.97
MAD	0.85	0.91	0.14	0.13
Bovine stomach				
NCC	0.56	0.66	0.54	0.94
MAD	0.27	0.26	0.75	0.12

It is clear from Fig. 2 that introducing the motion compensation approach significantly reduced motion artifacts in both the lens paper and bovine stomach reconstructions. The improvement in the MAD value for lens paper was a decrease from 0.85 to 0.13, and there was a decrease from 0.27 to 0.12 for the bovine stomach tissue. NCC scores increased from 0.44 to 0.97 for lens paper and from 0.56 to 0.94 for bovine stomach tissue. The mosaics also show optically sectioned images with reduced motion artifacts. As discussed below, cropping before mosaicking was sufficient to ensure residual artifacts were also reduced. In both cases, the combination of registration and pattern orientation correction yielded the greatest improvement over uncorrected reconstructions and reconstructions where only pattern orientation or registration was active.

However, some motion that cannot be corrected by this technique includes motion that occurs during the exposure. Effects from this occur as they do in SIM endomicroscopy, widefield endomicroscopy, and confocal endomicroscopy. In SIM endomicroscopy and widefield endomicroscopy, they appear as blur, and in confocal they appear as distortion. When features in a sample do not move with the probe, they may appear as repeated features as in Fig. 8, which, as can be seen, did not break the continuity of the mosaic.

Some artifacts toward the edges of the images were observed. These are seen as more pronounced residual grid patterns in the image periphery, as marked with an arrow in Fig. 8(d). The effect arises because, in a single widefield image, the intensity profile from out-of-focus depths is roughly uniform in the center of the probe but drops off near the edges. This is due to the effect of the edge of the bundle; out-of-focus depths nearer the edge receive less illumination than more central points. This does not matter for conventional SIM imaging as this is common to all three images, and the out-of-focus light is subsequently removed. However, it introduces a complication when reconstructing SIM images of a moving probe, as the three registered and shifted SIM images now have slightly different intensity profiles for the out-of-focus light contributions, leading to incomplete removal. These artifacts can be mitigated at the expense of a smaller FOV by cropping the raw images to the overlapping region with roughly uniform intensity. This has a similar effect to introducing compensating illumination surrounding a smaller diameter bundle. It may also be possible to computationally correct the raw images, which is a topic for further investigation.

Artifacts also occur when the motion of the probe is not perfectly parallel to the alignment of the patterns. The effects of slight misalignment caused by translation at an angle from parallel can be seen in Fig. 7(e) as streaks in the final mosaic. (These are unlikely to be edge artifacts as the cropping circle was set to half of the probe’s diameter where the widefield images had roughly uniform intensity.) The misaligment artifact partly arises due to the finite number of angles supported by the DMD, meaning that the direction of translation has to be rounded to the nearest supported angle.

Although the FOV of the individual motion-compensated moving SIM images is reduced, compared with conventional SIM there is no effective reduction of the usable FOV in any case. When the probe is stationary, the device performs as a conventional SIM; when the probe is in motion, a conventional SIM does not work at all. With motion compensation, the FOV is reduced and mosaicking is then possible, allowing for a larger effective FOV.

Other artifacts include repeated features in the freehand mosaics for bovine stomach. These were not observed in the tissue paper study, and so a likely cause is misregistration of tissue deformed during translation of the probe in contact mode (only rigid transformations were allowed for by the algorithm). Additionally, the latency associated with pattern selection meant that pattern orientation was always one acquisition sequence behind, leading to artifacts when the direction of travel changed between acquisition sequences. This is mostly caused by software limitations. Although template matching was benchmarked faster than the maximum camera frame rate (60 Hz), the updated angle was still received by the Pyboard shortly after the next sequence had started. Another source of latency was the speed at which LabVIEW determined the angle and sent the angle to the Pyboard via the DAQ. This could be reduced using a serial port to communicate with the Pyboard directly instead of analog pins, removing any delay in communication with the DAQ. A topic for further investigation is devising a scheme that would take advantage of both the fast template matching and serial data port to eliminate the pattern orientation lag.

With a mounted operation using a translation stage, in a configuration where angles do not need to be updated each cycle, artifacts caused by latency are mostly eliminated since the angle is the same for a large number of acquisition sequences. Similarly, handheld operation is more susceptible to artifacts resulting from deviations in movement parallel to line orientation. These would be reduced with a faster camera and DMD pattern selection time.

The frame rate of 2.5 fps was due to the lower duty cycle at higher frame rates forcing the camera exposure to be reduced. This arises from use of a low-cost off-the-shelf DMD with an onboard LED, which, without adding active cooling, had optical power limits and a DMD controller that does not allow patterns to be selected arbitrarily. Unfortunately, as each pattern has a minimum display time of 325  μs, as the frame rate is increased, the duty cycle decreases due to an increased fraction of the overall time needed to wait while unwanted frames are skipped. Pattern selection time and optical power are not fundamental limitations of this approach. Pattern selection time could be increased with a more sophisticated DMD controller that allowed selection of arbitrary patterns. The power at the fiber tip was measured to be 95  μW, and the fiber coupling losses were measured to be 89%. Power could be increased by adding active cooling to the DMD, optimizing lost light at the objective/bundle interface, or exchanging the DMD’s built-in lens and LED with a high-power light source.

## 4 Conclusion

Endomicroscopy is a useful tool for point-of-care diagnosis of epithelial cancers. A low-cost fiber bundle-based-SIM endomicroscope can be built with a probe area much less than the area of a conventional biopsy, motivating the need for mosaicking. During the three-frame SIM acquisition sequence, motion between raw frames can generate artifacts, which limit mosaicking. The findings presented here show that by reorienting patterns to the direction of motion, and by registering and shifting the images, it is possible to greatly reduce these artifacts. This will further advance the clinical relevance of SIM-based endomicroscopy.
